# Comparative analysis of the gut microbiota of hornbill and toucan in captivity

**DOI:** 10.1002/mbo3.786

**Published:** 2018-12-27

**Authors:** Cheng‐He Sun, Hong‐Yi Liu, Yong Zhang, Chang‐Hu Lu

**Affiliations:** ^1^ College of Biology and the Environment Nanjing Forestry University Nanjing China

**Keywords:** great hornbill, gut microbiota, toco toucan, wreathed hornbill

## Abstract

Gut microbiota plays an important role in animals and are considered microbial organs. Hornbill and toucan are birds of the same ecotypes with high appreciative value. In this study, we characterized and compared the gut microbiota of toco toucan (*Ramphastos toco*), great hornbill (*Buceros bicornis*) and wreathed hornbill (*Rhyticeros undulatus*) using 16S rRNA high‐throughput sequencing technology, and further discussed the influence of host bird genetics on its gut microbiota. We identified 10,847 operational taxonomic units (OTUs) from the hyper‐variable V4–V5 region, representing 14 phyla that were dominated by the Firmicutes, Proteobacteria, Cyanobacteria, Fusobacteria, Actinobacteria, and Bacteroidetes. Alpha diversity indices showed no significant difference among the three species (*p* > 0.1). However, great hornbill and toco toucan shared a high number of OTUs. Principal component analysis also revealed highly similar gut microbiotas between the two distant species. Therefore, environmental factors might dominate over host genetics in shaping the gut microbiotas of hornbill and toucan. Our study would contribute in elucidating adaptation of the hornbill and toucan to environmental change.

## 1 INTRODUCTION

Gut microbiota plays a crucial role in animals. Gut microbiotas are considered “microbial organs” of the living body (Clarke et al., 2014; Lyte, 2010). Immunity, nutrition, metabolism, and many other physiological functions are closely linked to gut microbiota (Kogut, 2013; Wu et al., 2017; Yuan et al., 2018). Birds represent a highly evolved lineage and play a significant role in ecosystem functioning (Waite & Taylor, 2015). Therefore, there have been many studies on the gut microbiota of avians (Grond, Sandercock, Jumpponen, & Zeglin, 2018; Hird, Sánchez, Carstens, & Brumfield, 2015; Ruizrodríguez, Lucas, Heeb, & Soler, 2009; Waite & Taylor, 2014). Previous studies have been diverse, with a large proportion of them investigating species, such as chickens, with economic value (Ding et al., 2017; Cross et al. (2007); Yang, Iji, & Choct, 2009) and red‐crowned crane, with high conservation value (Xie et al., 2016).

Both hornbills (Bucerotidae) and toucans (Ramphastidae) have distinctive beaks, making them the world's most recognizable birds. They inhabit the rainforest and are important seed dispersers; thus, they are considered key species (Whitney et al., 1998; Whitney & Smith, 2010). Hornbills are tropical and subtropical birds found mainly in Africa and Southern Asia. These birds are typical Scansores (Holbrook & Smith, 2000; Kemp, 2001; Trail, 2007). The toucan resembles the hornbill, and it is distributed in semi‐open habitats throughout a large part of central and eastern South America (Ragusa‐Netto, 2006). As ornamental birds, hornbill and toucan are common found species in zoos around the world (Seki, Bodde, & Meyers, 2010). Currently, both hornbill and toucan are listed as protected species under the Convention on International Trade in Endangered Species of Wild Fauna and Flora guidelines, due to habitat loss and human hunting.

In recent years, several researchers have conducted ecological and molecular studies on the hornbill and toucan, respectively (Gonzalez, Sheldon, Collar, & Tobias, 2013; Sammler, Bleidorn, & Tiedemann, 2011; Tattersall, Andrade, & Abe, 2009). To our knowledge, the genetic evolution of gut microbiotas of the hornbill and toucan has not been studied yet. Here, we characterized and compared the gut microbiota of the two bird species, and further discussed the influence of host bird genetics on gut microbiota. This study would elucidate the adaptation of the hornbill and toucan to environmental change.

## 2 MATERIALS AND METHODS

### 2.1 Fecal sample collection and preservation

All of the samples were collected from the Nanjing Hongshan Forest Zoo. Two species from the hornbill family—great hornbill (*Buceros bicornis*) and wreathed hornbill (*Rhyticeros undulatus*)—and one species from the toucan family—toco toucan (*Ramphastos toco*)—were selected. A total of 12 fecal samples from 12 birds aged 3–4 years were collected, with four samples per species. The three species of birds were housed in three different cages. They were fed at a fixed time each day with the same composition of food (no difference from the day of sampling), including rice rolls (rice, beef, eels, eggs, and carrots, cooked separately), bananas, cherries, tomatoes, grapes, and watermelon. Specifically, the individuals were fed at approximately 9 a.m., and they were monitored for fresh defecation within an hour. The collected feces were immediately snap‐frozen in liquid nitrogen, and then transported to the laboratory in dry ice.

### 2.2 DNA extraction

Total bacterial genomic DNA samples were extracted from the fecal samples using a QIAamp DNA Stool Mini Kit (QIAGEN, Germany) according to the manufacturer's instructions. The extracted DNA was examined using the Nanodrop spectrophotometer for quality, Qubit spectrophotometer for concentration, and agarose gel electrophoresis for integrity. The samples meeting the desired conditions were selected for sequencing.

### 2.3 16S rRNA amplicon pyrosequencing

PCR amplification of the bacterial 16S rRNA genes in the V4‐V5 region was performed using specific primers (forward primer 515F: 5′‐GTGCCAGCMGCCGCGGTAA‐3′, reverse primer 907R: 5′‐CCGTCAATTCMTTTRAGTTT‐3′). 16S rRNA gene sequencing was carried out by using a MiSeq platform. Sequencing was performed using an Illumina MiSeq platform with a MiSeq Reagent Kit v3 from Shanghai Personal Biotechnology Co., Ltd. (Shanghai, China). The high‐quality sequences were selected for subsequent analysis after the data were processed by quality control, splicing, filtering, and removal of chimeras.

### 2.4 Bioinformatics and statistical analysis

The Quantitative Insights Into Microbial Ecology (QIIME, v1.8.0) pipeline was employed to analyze the sequencing data obtained above (Caporaso et al., 2010). After chimera detection, the clustering of operational taxonomic units (OTUs) was carried out with a sequence similarity of 97% and evaluated based on the different classification levels of the obtained sequences (Edgar, 2010). A Venn diagram was generated to visualize the shared and exclusive OTUs among species groups using R based analysis of the occurrence of OTUs across groups regardless of their relative abundance (Zaura, Keijser, Huse, & Crielaard, 2009).

Operational taxonomic units‐level alpha diversity indices, including the Chao1, abundance‐based coverage estimator (ACE), Shannon, and Simpson index, were computed based on the OTU table in QIIME. One‐way ANOVA was used to identify significant differences in the alpha diversity indices among different species groups. Beta diversity analysis was performed to examine the structural variation of microbiota. Statistical analysis was done using Student's *t* test, and principal component analysis (PCA) was executed based on the genus‐level compositional profiles (Ramette, 2007). Linear discriminant analysis (LDA) effect size (LEfSe) method was used to identify genera differentially distributed among the three species groups (Segata et al., 2011). All statistical analyses were performed using the software SPSS 19.0, with a significance level of *p* < 0.05, and OriginPro 9.1, with partial result display.

## 3 RESULTS

### 3.1 Pyrosequencing data and OTU classification

**Figure 1:**
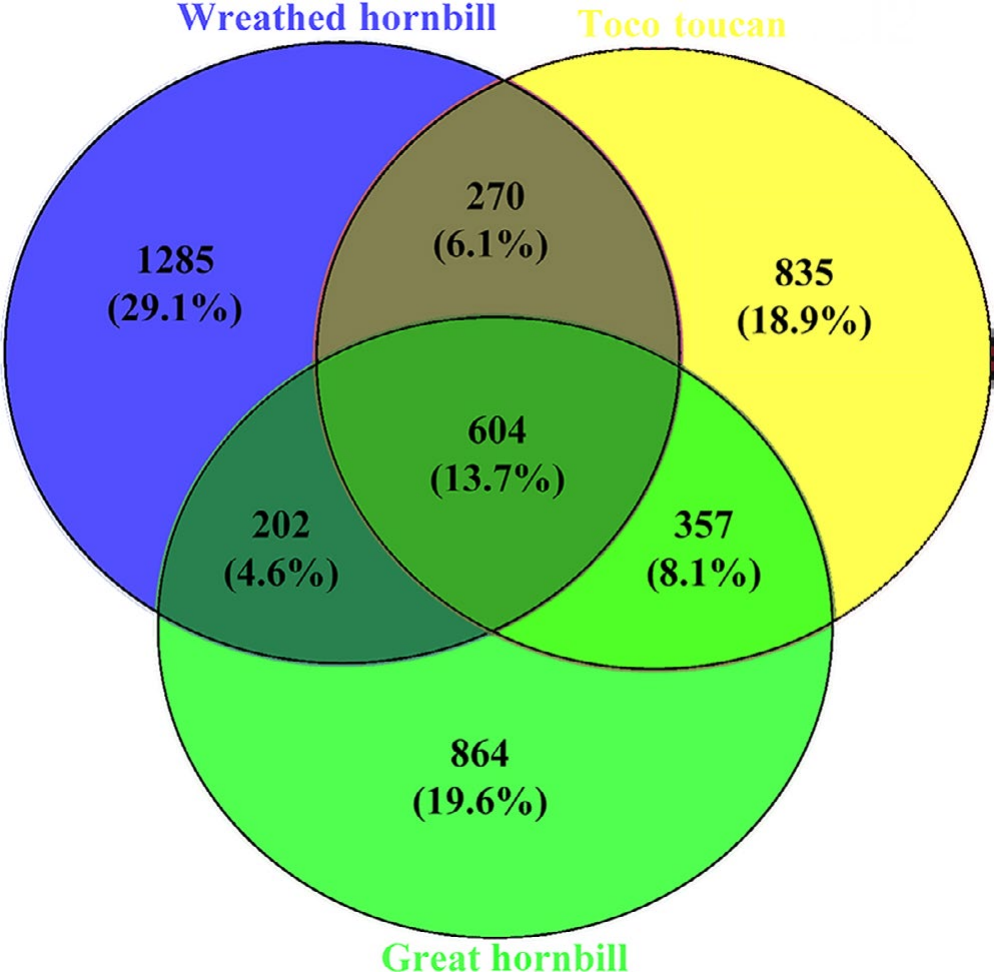
Venn diagram showing the number of shared operational taxonomic units among the three species groups

A total of 506,852 high‐quality sequences from the hyper‐variable V4‐V5 region of the 16S rRNA genes were obtained. This resulted in a total of 10,847 OTUs with 97% sequence similarity from a total of 14 phyla and 177 genera. In the three species, the actual number of OTUs was 2,066, 2,361 and 2,027 for toco toucan*,* wreathed hornbill, and great hornbill, respectively. As shown in Figure 1, 604 OTUs (13.7%) were shared among the three species groups, whereas 829 OTUs (18.8%) were detected in two out of the three species. Moreover, 2,984 OTUs (67.6%) were detected in only one species. To be specific, 202 OTUs (4.6%) were only shared between wreathed and great hornbill, 270 OTUs (6.1%) were only shared between wreathed hornbill and toco toucan, and 357 OTUs (8.1%) were only shared between great hornbill and toco toucan.

### 3.2 Diversity of gut microbiotas

Alpha diversity indices, estimates of species richness, and diversity are shown in Table 1 **.** In the three species, the alpha diversity indices for wreathed hornbill, great hornbill, and toco toucan were Chao1 (916, 928, and 1,016), ACE (944, 1,018, and 1,027), Shannon (6.03, 6.68, and 6.89) and Simpson index (0.91, 0.96, and 0.96), respectively. The great hornbill and toco toucan showed high similarity in alpha diversity indices as observed by the pairwise comparison. The alpha diversity indices of the great hornbill and toco toucan were more similar than those of two species of hornbills. However, there was no significant difference in alpha diversity indices among the three species (*p* > 0.05).

**Table 1:** Estimated species richness and diversity indices for the gut microbiota

Species group	Chao 1	Abundance‐based coverage estimator	Shanon	Simpson
Wreathed hornbill	916 ± 105	944 ± 111	6.03 ± 0.58	0.91 ± 0.03
Great hornbill	982 ± 53	1,018 ± 54	6.68 ± 0.35	0.96 ± 0.01
Toco toucan	1,016 ± 132	1,027 ± 124	6.89 ± 0.42	0.96 ± 0.01

Notes

Values of richness and diversity indices did not differ significantly among three species groups (*p* > 0.05). Values are shown as least squares means ± *SEM*.

### 3.3 Complexity of gut microbiotas at different level

**Figure 2:**
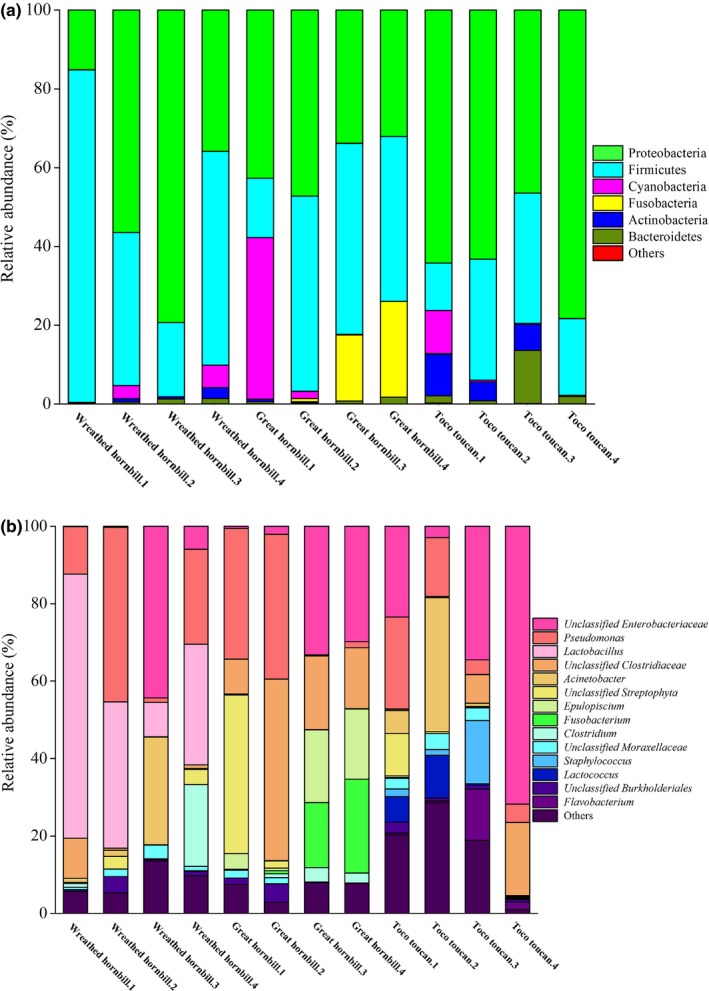
Main composition of the gut microbiota at phylum (a) and genus (b) level in the three species

The gut microbiota of each bird showed a mean of 904 ± 30 OTUs and was dominated by three phyla: Proteobacteria (49.6 ± 12.3%), Firmicutes (37.2 ± 12.7%), and Cyanobacteria (5.3 ± 4.7%). Across species, all birds exhibited relatively similar and overlapping gut microbiota (Figure 2a). Nine phyla were identified in the wreathed hornbill microbiota, with the majority of the sequences classified as either Firmicutes (18%–84%) or Proteobacteria (15%–79%). Nine phyla were identified in the great hornbill microbiota, with the majority of the sequences classified as Firmicutes (15%–49%), Proteobacteria (32%–47%), Cyanobacteria (0%–41%), or Fusobacteria (0%–24%). However, 14 phyla were identified in the gut microbiota of toco toucan, with the majority of the sequences classified as either Proteobacteria (46%–78%), Firmicutes (12%–33%), Cyanobacteria (0%–10%), Actinobacteria (0%–10%), or Bacteroidetes (0%–13%).

At the genus level (Figure 2b), a high percentage of the 16S rRNA sequences from the three species belonged to unclassified genera (43.0 ± 24.2%). The microbiota of wreathed hornbill was dominated by unclassified Enterobacteriaceae (0.1%–44%), *Pseudomonas* (1%–45%)*, Lactobacillus* (9%–68%)*, Acinetobacter* (0.3%–28%), and *Clostridium* (0%–21%). The microbiota of great hornbill was dominated by unclassified Enterobacteriaceae (0.5%–33%), *Pseudomonas* (0.2%–37%)*,* unclassified Clostridiaceae (9%–47%), unclassified Streptophyta (0%–41%), *Epulopiscium* (0.7%–19%), and* Fusobacterium* (0%–24%). The microbiota of toco toucan was dominated by unclassified Enterobacteriaceae (2.9%–72%), *Pseudomonas* (4%–24%)*,* unclassified Clostridiaceae (0%–35%), and* Acinetobacter* (0.2%–35%).

### 3.4 Differences in gut microbiotas

**Figure 3:**
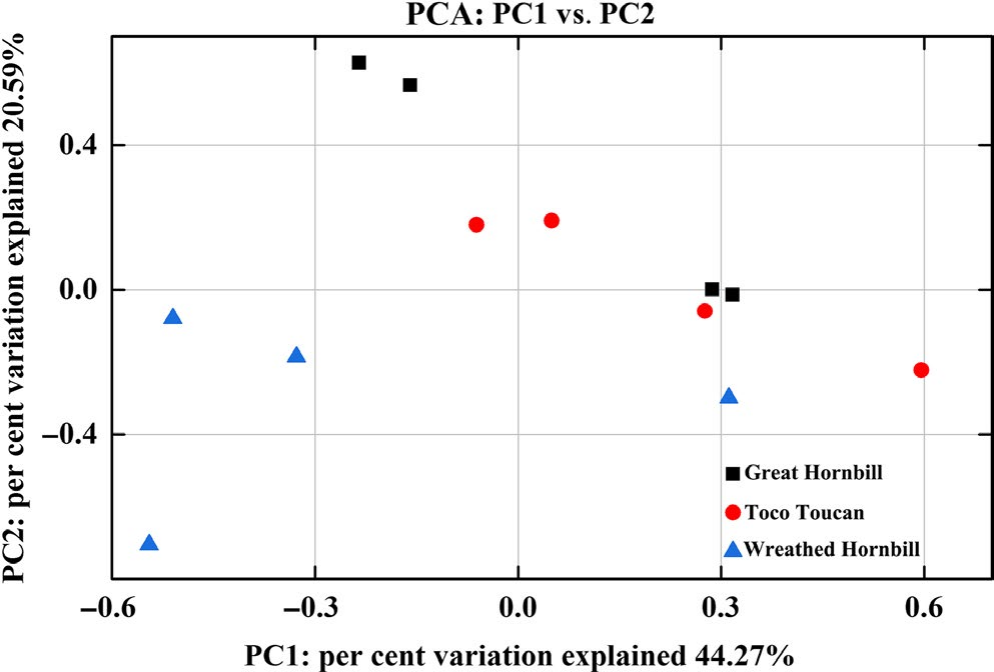
Principal component analysis (PCA) of operational taxonomic unit profiles (Bray–Curtis) of the three species. Red circles, black squares and blue triangles represent the gut microbiotas from the toco toucan, great and wreathed hornbill, respectively. Distances between symbols on the ordination plot reflect relative dissimilarities in community structures

**Figure 4:**
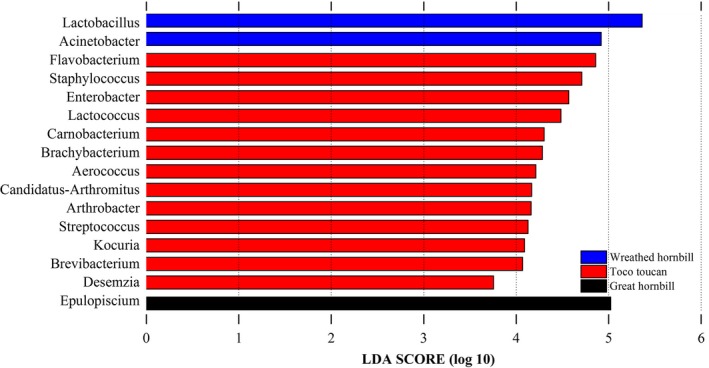
Genus differentially represented among the three species groups identified by linear discriminant analysis effect size

The PCA plot showed some differences among the three species. However, it also showed similarity between great hornbill and toco toucan (Figure 3). Among the three species groups, Actinobacteria was more abundant in toco toucan than in other species. At genus level, a total of 16 significantly differentially represented genera associated with each species group were identified using LEfSe. These genera mainly belong to the phyla Firmicutes (9/16), Actinobacteria (4/16) and Proteobacteria (2/16; Figure 4).

## 4 DISCUSSION

Birds are an integral part of all ecosystems and perform a variety of important functions such as seeds dispersion (Whitney et al., 1998), devouring harmful insects, and scavenging animal carrion (Wang et al., 2018). The current study is the first to assess the gut microbiota of hornbills and toucans. Firmicutes and Proteobacteria were the dominant phyla in the gut microbiotas of the three omnivorous species examined in this study, which corroborated with the previous studies on the gut microbiotas of omnivorous birds (Ding et al., 2017; Grond, Ryu, Baker, Domingo, & Buehler, 2014; Hird, Carstens, Cardiff, Dittmann, & Brumfield, 2014; Risely, Waite, Ujvari, Hoye, & Klaassen, 2018; Vecherskii et al., 2014). Furthermore, the Firmicutes and Proteobacteria were also found to be the most abundant bacterial phyla in the gut of other carnivorous birds (such as the Accipitridae vultures (Roggenbuck et al., 2014) and Spheniscidae penguins (Dewar et al., 2014)) and herbivorous birds (such as Anatidae geese (Wang et al., 2018) and Psittacidae Parrot (Waite, Eason, & Taylor, 2014)). The Firmicutes and Proteobacteria might be important for some physiological and biochemical functions of the gut of bird species. The important microbes might be passed down through the generations.

In addition to Firmicutes and Proteobacteria, we also found relatively high abundance of Cyanobacteria, Fusobacteria, Actinobacteria, and Bacteroidetes at the phyla level. The relatively high abundance of cyanobacteria was probably due to the chloroplasts extracted from the plant material in the food of great hornbills (Olsson, Gunnarsson, & Elmberg, 2017). Similar to the gut microbiotas of cowbirds (Hird et al., 2014), vultures (Roggenbuck et al., 2014), and penguins (Dewar et al., 2014), great hornbills also contained a relatively high abundance of Fusobacteria. The relatively high abundance of Fusobacteria in great hornbills resulted from the genus *Fusobacterium*, and it may be related to many physiological functions of gut (Hird, 2017). Actinobacterium is an abundant phylum in the gut microbiota of toucans, and studies have shown a positive correlation of actinobacteria with fiber intake in humans (Lee, Rusch, Stewart, Mattila, & Newton, 2015). Bacteroidetes have previously found in relatively high abundance in gut microbiotas of some birds, such as wild geese (Wang et al., 2018), cowbirds (Hird et al., 2014), and ostrich (Matsui et al., 2010), etc. This bacterium can help the host to get more plant nutrients.

The gut microbiotas of the great and wreathed hornbill were composed of nine phyla, and the gut microbiotas of the toco toucan were identified to be composed of fourteen phyla. This finding suggested that host genetics could play an important role in shaping the gut microbiotas of hornbill and toucans. At the genus level, we also found that the gut microbiotas differed greatly between toco toucan and the two hornbills. For example*,* the relative abundance of the genus *Brevibacterium* of the Brevibacteriaceae family in toucan was significantly higher than that in the two species of hornbills.

The species and relative abundance of gut microbiotas in the three species groups were similar (Figure 2). Moreover, the alpha diversity indices, Venn diagram, and PCA plot showed that there was more similarity in the gut microbiotas between great hornbill and toco toucan, while the microbiotas of great and wreathed hornbill were highly different from each other. These results might be due to the same feeding environment in the zoo. Therefore, the environmental factors have significant impacts on the gut microbiotas of hornbills and toucans.

## CONFLICT OF INTEREST

The authors declare that they have no conflicts of interest.

## AUTHORS CONTRIBUTION

CHL designed the research, HYL performed the experiments and revised the manuscript, YZ analyzed the data, and CHS wrote the manuscript. All authors read and approved the final manuscript.

## ETHICS STATEMENT

This research was approved by the ethics committee of Nanjing Forestry University and the veterinary hospital of Nanjing Hongshan Forest Zoo. This study did not involve any animal tissues. All fecal samples were collected by the keepers to avoid stress reaction of these birds.

## Data Availability

All data are included in the main manuscript. Raw data and materials are available on request.
